# Rumination and Default Mode Network Subsystems Connectivity in First-episode, Drug-Naive Young Patients with Major Depressive Disorder

**DOI:** 10.1038/srep43105

**Published:** 2017-02-22

**Authors:** Xueling Zhu, Qiuling Zhu, Huaizhen Shen, Weihua Liao, Fulai Yuan

**Affiliations:** 1 Health Management Center, Xiangya Hospital, Central South University, Changsha, 410008, China; 2 School of Humanities and Social Sciences, National University of Defense Technology, Changsha, 410074, China; 3 Medical Psychological Institute, Second Xiangya Hospital, Central South University, Changsha, 410011, China; 4 Obstetrics Department, Jinan Maternity and Child Care Hospital, Jinan, 250001, China; 5 Department of Radiology, Xiangya Hospital, Central South University, Changsha, 410008, China

## Abstract

Neuroimaging evidence implicates the association between rumination and default mode network (DMN) in major depressive disorder (MDD). However, the relationship between rumination and DMN subsystems remains incompletely understood, especially in patients with MDD. Thirty-three first-episode drug-naive patients with MDD and thirty-three healthy controls (HCs) were enrolled and underwent resting-sate fMRI scanning. Functional connectivity analysis was performed based on 11 pre-defined regions of interest (ROIs) for three DMN subsystems: the midline core, dorsal medial prefrontal cortex (dMPFC) and medial temporal lobe (MTL). Compared with HCs group, patients with MDD exhibited increased within-system connectivity in the dMPFC subsystem and inter-system connectivity between the dMPFC and MTL subsystems. Decreased inter-system connectivity was identified between the midline core and dMPFC subsystem in MDD patients. Depressive rumination was positively correlated with within-system connectivity in the dMPFC subsystem (dMPFC-TempP) and with inter-system connectivity between the dMPFC and MTL subsystems (LTC-PHC). Our results suggest MDD may be characterized by abnormal DMN subsystems connectivity, which may contribute to the pathophysiology of the maladaptive self-focus in MDD patients.

Rumination is defined as a mode of responding to distress that involves repetitively and passively focusing on symptoms of distress and on the possible causes and consequences of these symptoms^1^,^2^. It is strongly and consistently related to depressive symptoms^3^. Evidence from prospective longitudinal and experimental studies have emphasized the role of rumination in the onset and maintenance of symptoms and the diagnosis of major depressive disorder (MDD)^2^,^4^,^5^, onset of depressive symptomatology in non-depressed people^2^, and risk of depressive relapse in remitted patients^6^.

Although the pathophysiological mechanisms underlying MDD remain unclear, neuroimaging studies have shown that MDD has been conceptualized as a neural network-level disease^7^,^8^,^9^,^10^. Among the neural networks identified in MDD, the default mode network (DMN) has received growing attention. It encompasses a specific set of brain regions, including the medial prefrontal cortex (MPFC), precuneus/posterior cingulate cortex (PCC), and medial, lateral, and inferior parietal cortex^11^,^12^. Researchers have consistently reported that the DMN is involved in internal mentation, including self-referential processing, the memory retrieval process and autobiographical memory retrieval^13^,^14^,^15^, which is closely associated with depressive symptomatology^15^,^16^. Striking differences in the activity and functional connectivity of the DMN between individuals with MDD and healthy controls (HCs) have been identified in a large number of studies using either task-based^17^,^18^,^19^ or resting-state fMRI^20^,^21^,^22^,^23^. Despite inconsistent findings, the DMN has been considered to play a central role in the physiopathology of MDD^24^.

Recent imaging studies have suggested the intuitive association between the self-focused rumination in MDD and the self-referential operations performed by DMN^25^,^26^, which has prompted further interest in examining the role of DMN in MDD. It was revealed that people with MDD showed stronger functional connectivity with DMN in the subgenual prefrontal cortex (sgPFC), the degree of activation being modulated by the level of maladaptive rumination^27^,^28^. Similarly, our previous study also reported increased functional connectivity with DMN in anterior medial cortex including the sgPFC in MDD patients^29^. Furthermore, effective connectivity analysis revealed mutually propagating activation between the sgPFC and ventral MPFC in people with MDD, which predicted higher levels of depressive rumination^30^. Convergent findings from neuroimaging studies have identified the role of the sgPFC in the DMN and its relationship with rumination in MDD, which suggest that the sgPFC might be unique to depression and a neural substrate of depressive rumination^15^,^26^. However, the association between the rumination and DMN in MDD still remains unclear and requires further investigation in this field.

Although the DMN originally was regarded as a large-scale brain system, current studies have noted the DMN is not as homogenous as previously assumed and suggested that its architecture can be further subdivided into smaller anatomical–functional subsystems^31^. Furthermore, the subsystems and the interaction among them have been shown to provide interesting insights for exploring mental diseases^32^,^33^,^34^,^35^. Recent evidence both from monkeys and humans have suggested that the DMN comprises a set of interacting subsystems, including a midline core constituted by hub regions in the anterior medial prefrontal and posterior cingulate cortices as well as two functionally dissociable subsystems: the dorsal medial prefrontal cortex (dMPFC) subsystem involved in self-referential processes and the medial temporal lobe (MTL) subsystem involved in episodic memory^31^. Furthermore, altered interaction among the three subsystems within the DMN might be crucial in the psychopathology of some neuropsychiatric disorders, including Alzheimer’s disease^32^, obsessive-compulsive disorder^33^ and schizophrenia^34^. Previous studies have reported that patients with MDD exhibited abnormal connectivity in posterior, ventral and core DMN subsystems along with reduced interactions from the anterior to the ventral DMN subsystems^35^, which suggested the role of these subsystems and the interplay among them in the pathophysiology of MDD. To date, however, the extent to which altered function and interaction of three DMN subsystems (the midline core, dMPFC and MTL subsystems) can contribute to the pathophysiology of MDD is not known.

To the best of our knowledge, limited research has investigated neural correlates of rumination and three DMN subsystems in MDD. In the current study, our first aim was to investigate DMN subsystems connectivity in first-episode treatment-naïve young adults with MDD. The second goal was to examine the association between depressive rumination and DMN subsystems connectivity. We hypothesized that MDD patients would exhibit altered activity in DMN subsystems, which would correlate with depressive rumination.

## Results

### Demographic and clinical data comparisons

The demographic and clinical data were summarized in Table 1. There was no significant difference between the MDD and HCs groups in gender, age and years of education (Table 1). Compared with HCs group, MDD patients showed higher levels of Center for Epidemiologic Studies Depression (CES-D) (t = 10.030, p < 0.001) and rumination of Response to Stress Questionnaire (RSQ-rumination) (t = 6.980, p < 0.001).

### Differences in functional connectivity in 11 regions of interest (ROIs)

**Figure 1:**
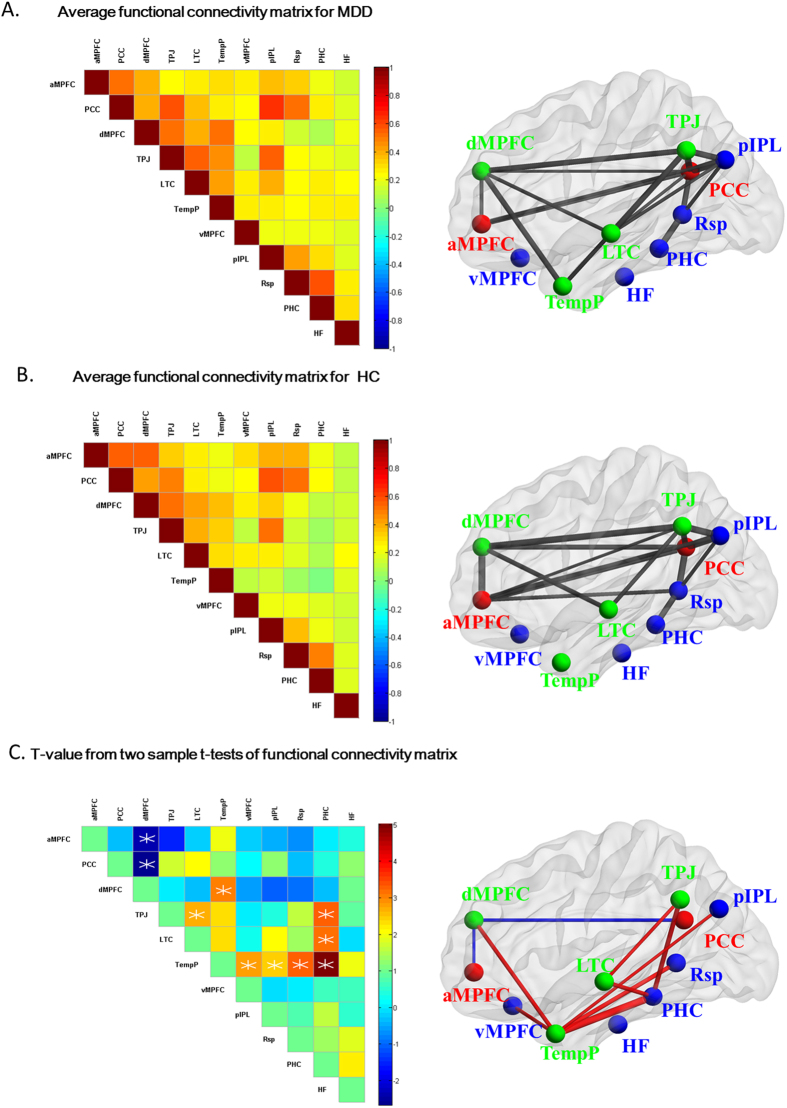
The left panels of (**A**) and (**B**) shows half of the averaged connectivity matrices among 11 ROIs of the DMN for MDD patients (**A**) and HCs group (**B**). The color bar indicates the value of correlation strength. The right panels of (**A**) and (**B**) showed visualized brain network for the averaged correlation coefficient matrix threshold by 0.35, where the thickness of each line reflects correlation strength. The left panel of (**C**) shows the two-sample t-tests of functional connectivity matrix in MDD compared with HCs group, where the white “*” denotes significantly altered connectivity (p < 0.05 with false discovery rate (FDR) correction). The color bar indicates t value. The right panel of (**C**) shows visualized brain network for the two-sample t-tests, where the red and blue lines respectively show increased and decreased functional connectivity, and the thickness of each line reflects t value.

The average half of 11 × 11 functional connectivity matrices is displayed for both MDD (Fig. 1A) and HCs (Fig. 1B) groups with a threshold of 0.35^34^. The MDD patients showed similar connectivity patterns among 11 ROIs as HCs group. As illustrated in Fig. 1C, compared with HCs, MDD patients showed increased connectivity in dMPFC-TempP, TPJ-LTC, TPJ-PHC, LTC- PHC, TempP-vMPFC, TempP-pIPL, TempP-Rsp and TempP-PHC (p < 0.05 with false discovery rate (FDR) correction). Of all, the dMPFC-TempP and TPJ-LTC were within-system connectivity in the dMPFC subsystem, whereas the others were inter-system connectivity between the dMPFC and MTL subsystems. Meanwhile, the decreased inter-system interactions were reported in MDD patients between the midline core and dMPFC subsystems, including aMPFC-dMPFC and PCC-dMPFC (p < 0.05 with FDR correction).

### Differences in functional connectivity of DMN subsystems

**Figure 2:**
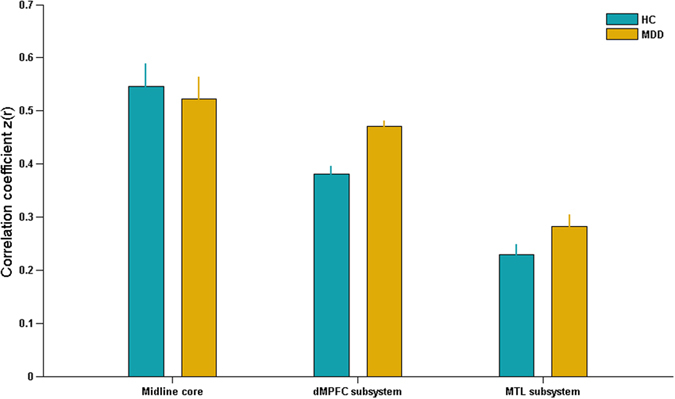


**Figure 3:**
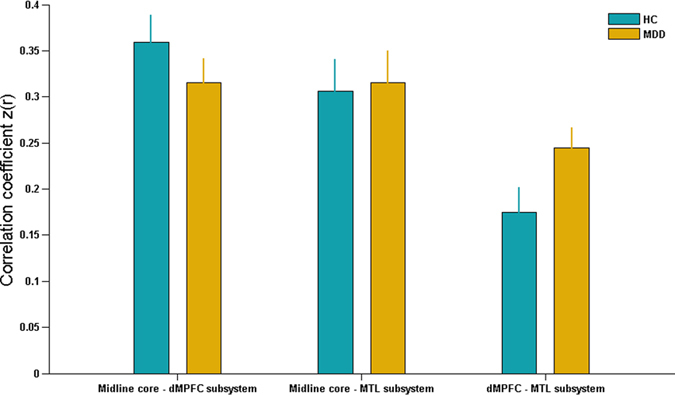


The average within-system and inter-system connectivity of DMN subsystems were respectively exhibited in Figs 2 and 3 in MDD and HCs groups. Relative to HCs group, the patients with MDD exhibited significantly increased within-system connectivity in dMPFC subsystem (t = 5.155, p < 0.001), but not in the midline core (t = 0.408, p = 0.685) or MTL subsystem (t = 1.835, p = 0.072). Additionally, the significantly increased inter-system connectivity of DMN subsystems was reported in dMPFC-MTL interaction in MDD patients (t = 2.039, p = 0.048), but not in the midline core-dMPFC (t = 1.126, p = 0.267) or midline core-MTL interaction (t = 0.195, p = 0.846).

### Correlation analysis

**Figure 4:**
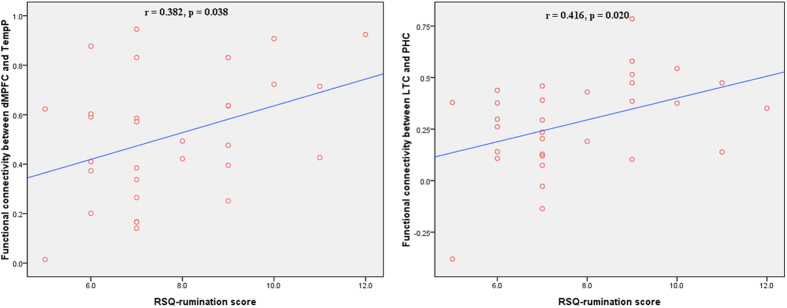


After controlling for CES-D score, age, education and illness duration as covariates, RSQ-rumination scores were positively correlated with functional connectivity in patients with MDD, including dMPFC-TempP (r = 0.382, p = 0.038) and LTC-PHC (r = 0.416, p = 0.020) (Fig. 4).

## Discussion

To date, few studies have focused on the neural activity of DMN subsystems in MDD patients, although previous work has investigated different aspects of the DMN. The present study aimed to investigate three DMN subsystems resting-state functional connectivity and the association with rumination in patients with MDD. Four principal findings emerged from our study. Firstly, we found significantly increased within-system connectivity in the dMPFC subsystem in patients with MDD (dMPFC-TempP and TPJ-LTC). Secondly, the increased inter-system connectivity was revealed between the dMPFC and MTL subsystems in MDD patients (TPJ-PHC, LTC-PHC, TempP-vMPFC, TempP-pIPL, TempP-Rsp and TempP-PHC). Thirdly, the decreased connectivity was observed between the midline core and dMPFC subsystem in individuals with MDD (aMPFC-dMPFC and PCC-dMPFC). Finally, correlation analysis revealed that the connectivity of dMPFC-TempP and LTC-PHC were positively correlated with depressive rumination in the MDD group.

In present study, we found significantly greater connectivity in the dMPFC subsystem in MDD group, including dMPFC-TempP and TPJ-LTC. These observations might reflect resting-state hyperconnectivity in the dMPFC subsystem in MDD patients. The dMPFC subsystem is thought to play more of a social-reflective role, allowing individuals to infer the mental states of other people and reflect on their own mental states^14^,^31^,^36^. In particular, as the key brain region in Mayberg’s model of MDD^37^,^38^, the dMPFC reportedly is involved in the process of evaluating whether stimuli are self-referential^39^,^40^,^41^ and is linked to a variety of cognitive functions, appraisal and the expression of negative emotion^42^. The neuropathological and neurochemical studies in major depression consistently reported abnormalities in the dMPFC subsystem^43^,^44^,^45^. Our findings of positive correlations between the rumination score and connectivity of dMPFC-TempP and LTC-PHC further support the view that the dMPFC subsystem is involved in self-abnormalities in MDD. Our study provides novel evidence of the notion that the dMPFC subsystem abnormalities in MDD might reflect the ruminative nature of MDD patients and further suggests that the resting-state signal in the dMPFC subsystem may be a marker for a ruminative response style in depression.

Furthermore, increased inter-system connectivity was observed between the dMPFC and MTL subsystems in MDD patients relative to HCs. It has been suggested that these two subsystems interact when individuals are left to think alone and undisturbed^46^. In such cases, people tend to engage in self-relevant internal cognitive processes, predominantly about significant past and future events^31^. The increased inter-system connectivity between the dMPFC and MTL subsystems in our study showed stronger self-relevant neural activity in MDD patients, which might be related to self-abnormalities in MDD. Consistent with this hypothesis, the following correlation results indicated that a positive correlation existed between LTC-PHC connectivity with rumination score. As far as we know, no study has investigated the relationship between rumination score and interaction within the dMPFC and MTL subsystems in subjects with MDD. Our findings provide new pathophysiological evidence that the interaction between the dMPFC and MTL subsystems may contribute to the ruminative response style in MDD, which is consistent with the previous studies mentioned above^46^. Our results support the notion that abnormal interaction between DMN subsystems might be involved in the pathophysiology of self-relevant symptoms in MDD.

In MDD patients, we detected decreased connectivity between the midline core and dMPFC subsystem, including aMPFC-dMPFC and PCC-dMPFC. Evidence from anatomical and imaging studies has revealed that both aMPFC and PCC have extensive connectivity with the dMPFC subsystem^47^,^48^. It has been suggested that midline core is critical to making self-relevant, affective decisions, which is strongly correlated with the dMPFC subsystem, especially in affective self-referential cognition^31^. The current finding of decreased inter-system connectivity might indicate impaired interaction between the midline core and dMPFC subsystem. It is worthy of mention that the result relating to the diminished connectivity of PCC-dMPFC is consistent with our previous study on first-episode treatment-naïve young adults with MDD^49^. The results were corroborated by other studies on resting-state connectivity in MDD^50^, suggesting decreased interaction between the anterior and posterior DMN in young people with depression. Nevertheless, increased PCC-dMPFC connectivity has been reported in late-life depression^51^,^52^,^53^, which implies that there is a relationship between PCC-dMPFC connectivity and patient age and medication status. Taken together, all the findings suggest that the pathophysiology of MDD could also be related to reduced central role of the midline core subsystem in the DMN.

The results of this study must be interpreted in light of its limitation. Firstly, as we selected first-episode, medication-naïve young patients with MDD to minimize the confounding influences of chronicity, treatment or comorbidity, the relatively small sample size may limit the generalizability of our results as well as our ability to detect more relationships between clinical variables and neuroimaging findings. Secondly, consistent with previous work^31^, seed-based analysis with the predefined 11 ROIs was used to calculate DMN subsystems connectivity. However, defining DMN based on data-driven approaches is also worth studying^54^. Finally, the acquisition parameters with large slices (4 + 1 mm), relatively short scanning time and high TR may have effect on the consistency of resting-state findings. More optimized acquisition parameters deserve investigation in future work.

## Conclusions

In summary, the present study examined the relationship between DMN subsystems connectivity and rumination in patients with MDD. Relative to HCs MDD patients demonstrated disrupted communication within DMN subsystems, which supported and extended the widely acknowledged large-scale network dysfunction in MDD^55^,^56^. The MDD patients showed increased within-system connectivity in the dMPFC subsystem, decreased inter-system connectivity between the midline core and dMPFC subsystem, and increased inter-system connectivity between the dMPFC and MTL subsystems. Importantly, abnormal connectivity in DMN subsystems had a significant positive correlation with rumination scores in MDD patients. Our findings suggest that disrupted integration of DMN subsystems may be closely associated with the pathophysiology of MDD patients, which highlights the importance of DMN subsystems connectivity.

## Methods

### Participants

Patients with MDD were recruited from the psychiatric clinic at Second Xiangya Hospital of Central South University in Changsha, China. Patients with MDD were diagnosed according to the Structured Clinical Interview for DSM-IV^57^ by independent assessments of two psychiatrists. All of the patients were experiencing their first episode of depression and had never received medication. Closely matched healthy subjects were recruited through advertisements from several colleges in Changsha. All subjects were right-handed. The shared exclusion criteria for patients and control subjects included any major medical illnesses; clinical diagnosis of neurologic trauma; any history of psychiatric disorder in the control subjects or any history of psychiatric disorder, except major depression, in the MDD patients; any history of substance abuse or alcohol in the past 6 months; and any contraindications to imaging scanning. Finally, 33 patients with MDD and 33 matched HCs were recruited.

### Research ethics

The study protocol was approved by Second Xiangya Hospital of Central South University. Written informed consent was obtained from all participants prior to the study, which was approved by the Institutional Review Board of Second Xiangya Hospital of Central South University for Brain Research. The methods were conducted in accordance with relevant approved guidelines and regulations.

### Measures

#### Depression

Depressive severity was measured using the CES-D scale^58^, a 20-item self-report instrument to assess depressive symptoms in the general population. The Chinese version of the CES-D has been found to have high degrees of reliability and validity^59^. In this study, the internal consistency of the CES-D was good (Cronbach’s alpha = 0.93).

#### Rumination

Level of rumination was assessed using the rumination subscale of RSQ in Chinese version^60^,^61^, which focuses on the feelings and thoughts that are associated with negative events. The rumination subscale of the RSQ exhibited high degrees of reliability and validity in both English and Chinese versions.

### Data acquisition

Images were obtained using a Siemens Skyra 3T scanner with a standard head coil. Participants wore a standard head coil fitted with foam padding to minimize head movement and diminish scanner noise. During scanning, all participants were required to remain motionless, keep their eyes closed and systematically try not to think of anything. After scanning, the participants were asked about their statement during scanning.

Resting-state fMRI images were acquired with a single-shot, gradient-recalled echo-planar imaging sequence oriented parallel to the line of the anterior-posterior commissure. The following parameters were applied: repetition time = 2000 ms, echo time = 30 ms, flip angle = 80°, field of view (FOV) = 240 mm × 240 mm, matrix = 64 × 64, slice thickness = 4 mm, slice gap = 1 mm, number of slices = 32. For each participant, 216 volumes were obtained, and the scan lasted 432 s.

High-resolution 3-dimensional (3D) structural images were acquired using a T1-weighted, magnetization-prepared rapid gradient-echo sequence. The following parameters were applied: repetition time = 1900 ms, echo time = 2.01 ms, flip angle = 9°, FOV = 256 mm × 256 mm, matrix = 256 × 256, slice thickness = 1 mm, slice gap = 0 mm, and number of slices = 176.

### Data preprocessing

Image preprocessing was performed using the Data Processing Assistant for Resting-State fMRI (DPARSF) professional software^62^ (http://www.restfmri.net). For each individual participant, the first 10 functional images were excluded from analysis. Subsequent images were corrected by slice timing and realigned for head motion. Two MDD patients and one healthy subject were excluded because their translation or rotation exceeded ±1.5 mm ±1.5°. The individual T1-weighted structural images were coregistered to functional images. The transformed structural images were then segmented into gray matter, white matter, and cerebrospinal fluid and normalized to Montreal Neurologic Institute (MNI) space. These transformation parameters were also applied to the functional images. The normalized functional images were resampled at a resolution of 3 × 3 × 3 mm^3^ and spatially smoothed with a 6-mm full width at half maximum Gaussian kernel. The sources of spurious variance were regressed out including 6 parameters from head-motion correction (Friston 24-parameter model)^63^, white matter and cerebrospinal fluid signal. Finally, functional images with linear trend were removed by temporal bandpass filtering (0.01–0.08 Hz).

In view of the influence of head motion on functional connectivity results^64^,^65^,^66^, the data was further performed with the scrubbing method to remove time points affected by head motions. As recommended by Yan *et al*.^66^, the framewise displacement (FD) was calculated by translation and rotation parameters of head motion based on the formula from previous study^64^. The image frames (FD > 0.5 mm) were identified as bad time points. Along with the bad time points, 1 preceding and 2 following points were deleted to assure exclusion of motion-related confounds. No difference was observed for the mean FD between MDD and HCs groups (Mann Whitney U-test, P = 0.27). For all subjects, the remaining imaging data contained more than 128 volumes.

### Definition of 11 ROIs

**Figure 5:**
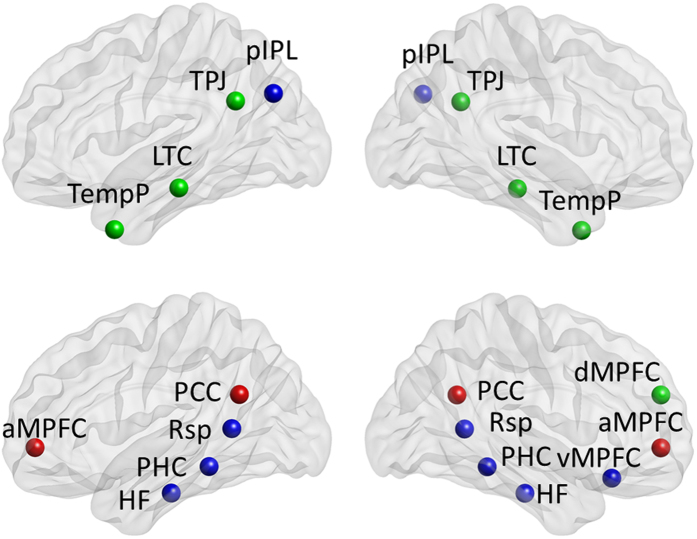
11 regions of interest (ROIs) in the default mode network (DMN) are depicted with BrainNet Viewer^70^, including the midline core denoted by red color, dMPFC subsystem denoted by green color, and MTL subsystem denoted by blue color. The midline core includes posterior cingulate cortex (PCC) and anterior medial prefrontal cortex (aMPFC). The dMPFC subsystem includes dorsal medial prefrontal cortex (dMPFC), temporo-parietal junction (TPJ), lateral temporal cortex (LTC) and temporal pole (TempP). The MTL subsystem includes ventral medial prefrontal cortex (vMPFC), posterior inferior parietal lobule (pIPL), retrosplenial cortex (Rsp), parahippocampal cortex (PHC) and hippocampal formation (HF).

According to previous work^31^,^32^,^33^,^34^, 11 ROIs of the DMN were defined using 8-mm radius spheres (see Fig. 5 and Table 2), including the anterior MPFC (aMPFC), PCC, dorsal MPFC (dMPFC), temporo-parietal junction (TPJ), lateral temporal cortex (LTC), temporal pole (TempP), ventral MPFC (vMPFC), retrosplenial cortex (Rsp), posterior inferior parietal lobule (pIPL), parahippocampal cortex (PHC) and hippocampal formation (HF). The aMPFC and PCC constitute the midline core. The dMPFC subsystem includes the dMPFC, TPJ, LTC and TempP. The MTL subsystem comprises the vMPFC, pIPL, Rsp, PHC and HF.

### Functional connectivity analysis

Functional connectivity analysis was performed on the left-lateralized 11 ROIs mentioned above (see Fig. 5 and Table 2) with the Resting-State fMRI Data Analysis Toolkit^63^. The mean time series from the voxels within each ROI were extracted. Pearson’s correlations were calculated between any two nodes of 11 ROIs. The correlation coefficients referred to the functional connectivity strength corresponding to the paired ROIs. The 11 × 11 functional connectivity matrix was obtained for each subject^68^,^69^. Fisher’s r-to-z transformation was applied to normalize the correlation coefficients. Apart from the individual edge corresponding to one paired ROIs, the average connectivity of all edges is another indicator to represent the global functional connectivity strength. Then, the average within-system and inter-system connectivity of three DMN subsystems for each subject were calculated^48^. The average within-system connectivity is defined as the mean of the sum of all edges in one subsystem, while the average inter-system functional connectivity is referred to the mean of the sum of functional connectivity strength of all edges between two subsystems.

### Statistical analysis

To examine group differences in functional connectivity of 11 ROIs, the two-sample t-tests were performed on half of 11 × 11 correlation coefficients matrices of MDD and HCs groups due to symmetry. The statistical significance level was set at p < 0.05 (FDR correction). The results with the undirected edges and 11 nodes were laid out in the BrainNet Viewer software^70^ (http://www.nitrc.org/projects/bnv/). To investigate group differences in average connectivity of three DMN subsystems, the two-sample t-tests were applied to explore the average within-system and inter-system connectivity between patients with MDD and HCs.

### Correlation analysis

To examine the association of DMN subsystems connectivity with the level of depressive rumination, linear correlations were calculated in MDD patients between the z-value of all edges and RSQ-rumination scores with SPSS 18.0 software (IBM SPSS Inc., USA). Given that these analyses were exploratory, we used an uncorrected statistical significance level of p < 0.05.

## Additional Information

**How to cite this article**: Zhu, X. *et al*. Rumination and Default Mode Network Subsystems Connectivity in First-episode, Drug-Naive Young Patients with Major Depressive Disorder. *Sci. Rep.* **7**, 43105; doi: 10.1038/srep43105 (2017).

**Publisher's note:** Springer Nature remains neutral with regard to jurisdictional claims in published maps and institutional affiliations.

## Figures and Tables

**Table 1:** Demographic and clinical characteristics for subjects.

Characteristic	MDD (n = 31)	HC (n = 32)	P value
Gender (F/M)	17/14	17/15	0.657^a^
Age (years)	20.53 ± 1.78	20.96 ± 1.28	0.434^b^
Education (years)	13.62 ± 0.76	13.75 ± 0.82	0.611^b^
Illness duration (years)	0.29 ± 0.20	NA	
CES-D	36.90 ± 6.79	17.97 ± 7.93	<0.001^b^
RSQ-rumination	7.74 ± 1.18	4.65 ± 1.68	<0.001^b^

MDD: major depressive disorder; HC: healthy control; Data value are Mean ± SD; F/M: female/male; SD. CES-D: Center for Epidemiologic Studies Depression; RSQ: Response to Stress Questionnaire. ^a^Chi-square test; ^b^Two sample t-test.

**Table 2:** Coordinates of 11 regions of interest (ROIs) in the default mode network (DMN).

DMN Subsystem Regions	Abbreviation	BA	MNI_xyz_
midline core			
Anterior medial prefrontal cortex	aMPFC	10, 32	−6, 52, −2
Posterior cingulate cortex	PCC	23,31	−8, −56, 26
dMPFC self system			
Dorsal medial prefrontal cortex	dMPFC	9, 32	0, 52, 26
Temporal parietal junction	TPJ	40, 39	−54, −54, 28
Lateral temporal cortex	LTC	21, 22	−60, −24, −18
Temporal pole	TempP	21,38	−50, 14, −40
MTL memory system			
Ventral medial prefrontal cortex	vMPFC	11, 24, 25, 32	0, 26, −18
Posterior inferior parietal lobule	pIPL	39	−44, −74, 32
Retrosplenial cortex	Rsp	29, 30, 19	−14, −52, 8
Parahippocampal cortex	PHC	20, 36, 19	−28, −40, −12
Hippocampal formation	HF	20, 36	−22, −20, −26

BA: Brodmann area; MNI: Montreal Neurological Institute; MTL: medial temporal lobe.
